# Referral and management patterns for paediatric otorrhoea across primary and secondary care: a multicentre retrospective cohort study

**DOI:** 10.1017/S1463423626101297

**Published:** 2026-06-16

**Authors:** Elliot Heward, Alexander Collingwood, Farah Jeffry, Shailesh Agrawal, Sharan Jayaram, Darren R. Ashcroft, Alastair D. Hay, Jaya R. Nichani, Iain A. Bruce

**Affiliations:** 1 University of Manchester School of Biological Science, UK; 2 Northern Care Alliance NHS Foundation Trust, UK; 3 Lancashire Teaching Hospitals NHS Foundation Trust, UK; 4 University of Manchester School of Pharmacy and Pharmaceutical Sciences, UK; 5 University of Bristol Department of Community Based Medicine, UK; 6 Royal Manchester Children’s Hospital, UK

**Keywords:** bacteriology, chronic otitis media, health services research, otitis media, primary care

## Abstract

**Aim:**

To determine the frequency and nature of referrals for children with acute or chronic otorrhoea from primary care to secondary care ENT services in the UK.

**Background:**

Middle ear infections in children are common; if the ear drum bursts discharge leaks out (otorrhoea). This causes hearing loss during a critical developmental period. Managing these children in an appropriate time frame to prevent disease repercussions is vital. There is currently no evidence demonstrating referral patterns and management strategies across primary and secondary care services.

**Method:**

Children with otorrhoea were identified amongst a cohort of 2,100 paediatric ENT (age 0–16 years) referrals from primary care at two secondary care hospital trusts in England in 2023. Chi-squared statistical analysis was performed to compare referral urgency for those with or without hearing loss.

**Findings:**

Of the paediatric ENT referrals, 228 (10.9%) had otorrhoea (mean age: 6.4 years, female: *n* = 110). The most frequent symptom duration at time of referral was >3–6 months (21.1%). Children with hearing loss were not referred more urgently compared to those without reported hearing loss (28.1% vs. 29.4%, *p* = 0.832). Antibiotic use in primary care was predominantly using oral antibiotics compared to topical antibiotics in secondary care. This study has shown that children with otorrhoea make up a significant proportion of paediatric referrals to the ENT secondary care services in the UK. Current management is heterogenous and could contribute to treatment failure. Standardized management pathways for these patients should be formulated.

## Introduction

Middle ear infections are common in children with 80% experiencing an infection before the age of three (Khairkar *et al*., 2023). Middle ear infections will cause discharge (otorrhoea) if the tympanic membrane bursts or in the presence of a pre-existing perforation. The terminology for paediatric otorrhoea has recently been outlined (Heward *et al*., 2025a). Acute ear discharge lasting up to 6 weeks is called acute otitis media with discharge (AOMd). Discharge lasting past this time point is called chronic suppurative otitis media (CSOM). Approximately 41,000 primary care appointments are required in the UK each year to manage children with AOMd or CSOM (Heward *et al*., 2025b).

Paediatric AOMd and CSOM can cause both temporary and permanent hearing loss which can lead to developmental delay (Khairkar *et al*., 2023). Other repercussions include child suffering, discomfort, social stigmatism due to cleanliness and financial cost to families (Heward *et al.*, 2024). Infection can spread to extracranial, intratemporal, and intracranial spaces, with these diagnoses prompting urgent admission to hospital via the emergency department.

Management of children with otorrhoea can be challenging. Primary care clinicians have described using the National Institute of Health and Care Excellence (NICE) guidance as their main management guide (Heward *et al*., 2024). NICE suggests treating AOMd with oral antibiotics (NICE, 2022a). NICE CSOM guidance recommends not attempting treatment, but to refer patients to secondary care. It defines CSOM as otorrhoea duration for greater than 2 weeks (NICE, 2022b). Parents have highlighted concerns about heterogenous treatment strategies and conflicting advice (Heward *et al*., 2024). Understandably, these conflicting guidelines cause clinician confusion.

Research is required to help standardize treatment guidelines. Before this is possible, we must understand the current landscape across primary and secondary care. This study aims to determine the referral frequency and management practice for children with AOMd and CSOM across primary and secondary care in the UK.

## Methods

The SQUIRE 2.0 framework was used for reporting this quality improvement project (Ogrinc *et al.*, 2016). A retrospective cohort study was performed at two secondary care hospital trusts in England, to identify all routine and urgent paediatric Ear, Nose, and Throat (ENT) outpatient referrals (age 0–16 years) from primary care from 1^st^ January 2023 to 31^st^ December 2023. Emergency admissions were not included. Patients with AOMd or CSOM were identified by reviewing the referral letter to identify whether otorrhoea was described. Referrals were reviewed for demographics, symptom duration, hearing loss, and prior management. The records from the patient’s first secondary care ENT appointment were then reviewed. Data that could not be retrieved was classed as missing data. Data was stored on excel on the respective NHS trust secure networks. Patient details were anonymized once data was collected.

The patient population that centre A and centre B serve is 670,000 and 395,000 people, respectively. Centre A received 1,096 and centre B 1,004 referrals during the study duration. Prolonged antibiotic course was defined as greater than two weeks duration.

Chi-squared statistical analysis was used to compare referral urgency by hearing loss (GraphPad 2024). This non-parametric test was selected to compare observed frequencies of two categorical variables in a two-by-two contingency table. It was also chosen due to the large sample number. Significant difference was defined as *p* < 0.05. Descriptive analysis was performed for all other data. Research ethics approval was not required according to the Health Research Authority. The study was registered with the audit departments at both hospitals.

## Results

**Figure 1.**
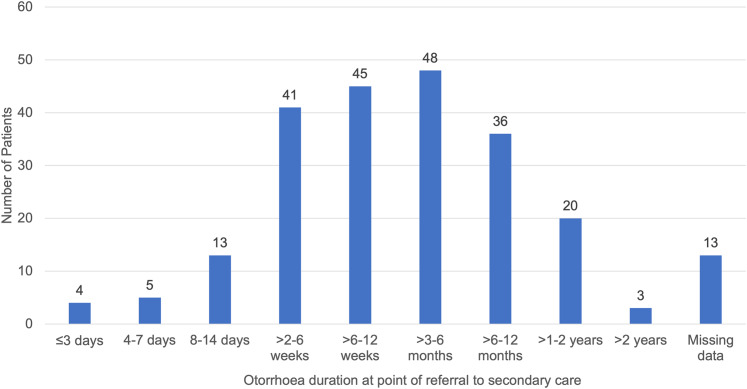
Figure 1 long description. Symptom duration prior to secondary care referral.

**Figure 2.**
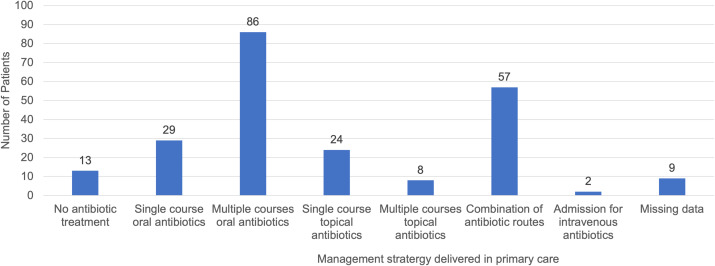
Figure 2 long description. Antibiotic treatment delivered in primary care prior to secondary care referral.

In total, 2,100 paediatric ENT referrals were received across both sites of which 228 (10.8%) had AOMd or CSOM (Table 1). Duration of symptoms ranged from <3 days to over 2 years at time of referral from primary to secondary care (Figure 1). Half of the patients were referred with perceived hearing loss. Referrals of children with AOMd or CSOM were marked as urgent by the GP in 28.1% (*n* = 64) of cases. Patients with hearing loss were not referred more urgently than those without hearing loss (28.1% vs. 29.4%, chi squared: 0.045, *p* = 0.832). The most frequent treatment children received prior to the secondary care referral was multiple courses of oral antibiotics (*n* = 86, 37.7%) (Figure 2).

**Table 1.** Patient demographics comparing all referrals to ENT versus those referred with otorrhoea Table 1 long description.

	All referrals	Referred with otorrhoea
*n*	2,100	228
Mean age (years)(range)	7.1 (0–16)	6.4 (0–16)
Female	973 (46.3%)	110 (48.2%)
Hearing loss at referral		
Yes	–	114 (50.0%)
No	–	109 (47.8%)
Missing data	–	5 (2.2%)

**Figure 3.**
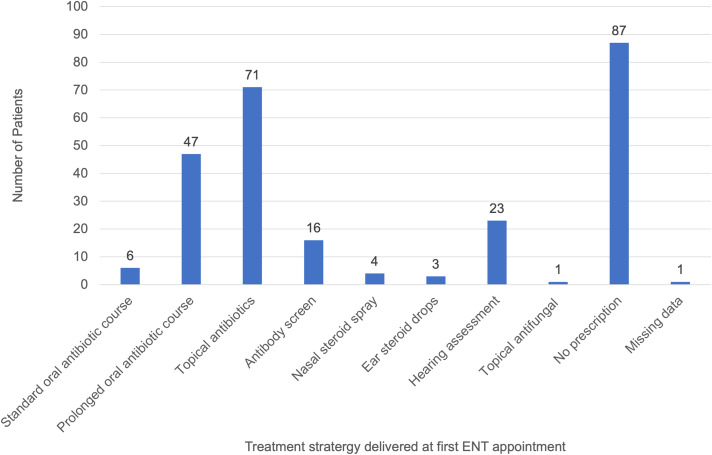
Figure 3 long description. Management strategy at initial secondary care ENT appointment.

**Figure 4.**
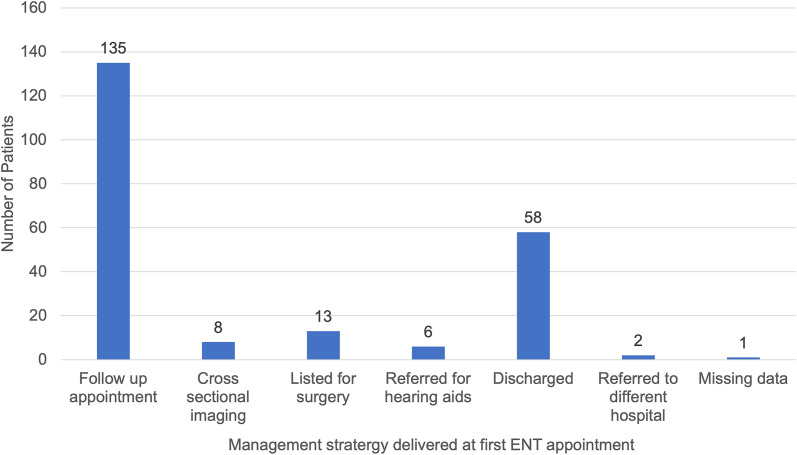
Figure 4 long description. Management plan following initial secondary care ENT appointment.

At the patient’s initial ENT appointment, the most frequent prescription was for topical antibiotics (*n* = 71, 31.1%) followed by a prolonged course of oral antibiotics (*n* = 47, 20.6%) (Figure 3). A large proportion of patients (*n* = 87, 38.2%) had no medication prescribed. The majority (*n* = 135, 59.2%) of patients were provided with a follow-up appointment (Figure 4). Thirteen patients were listed for surgery (5 grommets, 3 adenotonsillectomy with grommets, 1 adenotonsillectomy, 3 examination under anaesthetic, and 1 myringoplasty).

## Discussion

In 2017/2018, GPs in England made 900,000 adult and paediatric ENT referrals (NHS, 2019). This study has identified that 10.8% of paediatric ENT referrals from primary care were for children with AOMd or CSOM. Therefore, using the population ratio for adults and children in England in 2022, it can be approximated that 12,000 children are referred to secondary care services with otorrhoea each year (ONS, 2024). This demonstrates significant burden placed on secondary care services and the scale of initial treatment failure in primary care.

Otorrhoea duration at time of referral is variable. It is concerning that children are suffering with months of otorrhoea, potentially impacting hearing and development, before being referred. Children with persistent otorrhoea despite adequate medical management should be reviewed by ENT to rule out a cholesteatoma. Prolonged otorrhoea duration at referral could be explained by late presentation to primary care, unclear referral and treatment guidance, and a drive to reduce secondary care referrals (NHS, 2019). Families of children with otorrhoea have demonstrated high levels of health seeking behaviour due to parental concern, which makes delayed presentation less likely (Heward *et al*., 2024). In addition, in England under half of patients (49.2%) are being seen in secondary care ENT clinics within 18 weeks of referral, further delaying patient care (NHS, 2024). These cumulative factors result in management delay and increase the risk of permanent sequalae from ear infections.

Conductive hearing loss secondary to middle ear infections often occurs when children are in a key developmental period (NIDCD, 2022). It is postulated that toxins associated with middle ear infection can diffuse through the round window, potentially causing permanent cochlea damage (sensorineural hearing loss) (Dobrianskyj *et al*., 2017). It is unclear how frequently middle ear infection causes sensorineural hearing loss and the long-term repercussions (Elzinga *et al*., 2011). This research has identified that half of children referred with AOMd or CSOM had suspected hearing loss; with 28.1%, being referred to secondary care urgently. Interestingly, only 10.1% of children underwent an age-appropriate hearing test in secondary care (Figure 3). Reasons could include resolution of symptoms, lack of access to paediatric audiology or reluctance to test while otorrhoea is present. The results of this study suggest that hearing loss is not prioritized by GPs or ENT. In children it can be difficult to determine if unilateral hearing loss is present as children will compensate using their contralateral normal hearing ear. Future research is needed to examine the short- and long-term impact on hearing and development.

Globally, there is increasing concern about antibiotic overuse and antimicrobial resistance (AMR) (GOV, 2024). Previous work has shown that children with AOMd are predominantly managed with oral antibiotics in primary care (Heward *et al*., 2025b). The current study has demonstrated a higher frequency of oral antibiotic use compared to that in the literature (75.4% current vs 57.1%) (Heward *et al*., 2025b). This is due to an inherent selection bias because all included patients in our study required a secondary care referral suggesting infections refractory to standard treatment. Very few children were referred to secondary care without preceding antibiotic management.

There is currently no evidence outlining secondary care management of children with otorrhoea. Our results demonstrate variable management strategies including the use of topical or long-term oral antibiotics. Interestingly, 38.2% received no medication at their first secondary care appointment. This could be due to symptom resolution prior to review or subsequent treatment in primary care whilst awaiting secondary care review. The heterogenous antibiotic use seen in primary and secondary care settings mandates further research to help develop a standardized approach.

The majority of patients were arranged follow-up appointments after their initial secondary care review demonstrating the challenge in managing this patient population. Parental concern is known to influence the clinician’s decision-making (Heward *et al*., 2024).

There are limitations to this retrospective study such as the missing data which will skew the results. There is also likely to be regional variation in practice which will not be reflected in this work. Interestingly, there is a large difference between the ratio of paediatric ENT referrals to secondary care and the total population served comparing both centres in this study. Reasons could include the local age group mix, referral practices, access to secondary care services, access to GPs with specialist interest in ENT and local commissioning. Within our patient cohort, it was not possible to identify if the total symptom duration consisted of continuous or recurrent episodes of otorrhoea. Children could potentially have numerous shorter episodes which do respond well to treatment, but when referred the total duration of symptoms is outlined. The content of referrals was variable due to the free text nature of the referral templates. To enhance the data quality and to aid clinic prioritization electronic referral forms requiring key clinical information to be populated should be designed.

## Conclusion

Children with AOMd and CSOM make up over 10% of paediatric referrals to the ENT secondary care services, approximately 12,000 each year in England. The duration of symptoms at referral and management strategies in primary and secondary care are variable. A collaborative approach by primary and secondary care services is required to develop standardized clinical data collection and treatment pathways based on high level evidence.

## Data Availability

Data is available upon request to the corresponding author via email.

## Table 1. Long description

The table presents a comparison of patient demographics between all referrals to ENT and those specifically referred with otorrhoea. It consists of four rows and three columns. The columns are labeled ‘All referrals’, ‘Referred with otorrhoea’, and include various demographic metrics. The first row indicates the total number of referrals, with 2,100 for all referrals and 228 for those with otorrhoea. The second row shows the mean age and range, with all referrals having a mean age of 7.1 years (range 0-16) and those with otorrhoea having a mean age of 6.4 years (range 0-16). The third row details the number and percentage of female patients, with 973 (46.3%) for all referrals and 110 (48.2%) for those with otorrhoea. The fourth row focuses on hearing loss at referral, showing 114 (50.0%) with hearing loss, 109 (47.8%) without, and 5 (2.2%) with missing data for those referred with otorrhoea.

Navigate back to Table 1..

## Figure 1. Long description

The bar graph compares the number of patients with different durations of otorrhoea at the point of referral to secondary care. The x-axis represents the duration categories, including less than or equal to 3 days, 4-7 days, 8-14 days, greater than 2-6 weeks, greater than 6-12 weeks, greater than 3-6 months, greater than 6-12 months, greater than 1-2 years, greater than 2 years, and missing data. The y-axis represents the number of patients, ranging from 0 to 60. The bars are vertical and show the following data points: 4 patients for less than or equal to 3 days, 5 patients for 4-7 days, 13 patients for 8-14 days, 41 patients for greater than 2-6 weeks, 45 patients for greater than 6-12 weeks, 48 patients for greater than 3-6 months, 36 patients for greater than 6-12 months, 20 patients for greater than 1-2 years, 3 patients for greater than 2 years, and 13 patients for missing data. The color scheme is blue for all bars. The graph highlights that the highest number of patients, 48, have otorrhoea for greater than 3-6 months, while the lowest number, 3, have otorrhoea for greater than 2 years.

Navigate back to Figure 1..

## Figure 2. Long description

The bar graph compares different antibiotic treatment strategies delivered in primary care before secondary care referral. The x-axis lists the management strategies: No antibiotic treatment, Single course oral antibiotics, Multiple courses oral antibiotics, Single course topical antibiotics, Multiple courses topical antibiotics, Combination of antibiotic routes, Admission for intravenous antibiotics, and Missing data. The y-axis represents the number of patients, ranging from 0 to 100. The bars are vertical and show the following data: No antibiotic treatment (13 patients), Single course oral antibiotics (29 patients), Multiple courses oral antibiotics (86 patients), Single course topical antibiotics (24 patients), Multiple courses topical antibiotics (8 patients), Combination of antibiotic routes (57 patients), Admission for intravenous antibiotics (2 patients), and Missing data (9 patients). The highest number of patients received multiple courses of oral antibiotics, while the lowest number of patients were admitted for intravenous antibiotics.

Navigate back to Figure 2..

## Figure 3. Long description

The bar graph compares the number of patients receiving various treatment strategies at their first ENT appointment. The x-axis lists the treatment strategies, including standard oral antibiotic course, prolonged oral antibiotic course, topical antibiotics, antibody screen, nasal steroid spray, ear steroid drops, hearing assessment, topical antifungal, no prescription, and missing data. The y-axis represents the number of patients, ranging from 0 to 100. The graph features vertical bars in blue, with the highest bar representing topical antibiotics with 71 patients, followed by no prescription with 87 patients. Other notable treatments include prolonged oral antibiotic course with 47 patients, hearing assessment with 23 patients, and antibody screen with 16 patients. The remaining treatments have significantly fewer patients, with nasal steroid spray at 4, ear steroid drops at 3, topical antifungal at 1, and missing data at 1. The graph highlights the prevalence of topical antibiotics and no prescription as the most common treatment strategies.

Navigate back to Figure 3..

## Figure 4. Long description

The bar graph compares the number of patients with different management strategies delivered at their first ear, nose, and throat appointment. The x-axis represents the management strategies, including follow-up appointment, cross-sectional imaging, listed for surgery, referred for hearing aids, discharged, referred to a different hospital, and missing data. The y-axis represents the number of patients, ranging from 0 to 160. There are seven vertical bars, each representing a different management strategy. The tallest bar, representing follow-up appointments, reaches 135 patients. The second tallest bar, representing discharged patients, reaches 58 patients. Other bars are significantly shorter, with values of 13 for listed for surgery, 8 for cross-sectional imaging, 6 for referred for hearing aids, 2 for referred to a different hospital, and 1 for missing data. The color scheme uses blue for all bars. The graph highlights that the most frequent management strategy is scheduling a follow-up appointment, followed by discharging patients.

Navigate back to Figure 4..
